# Supplementary material for: DMRforPairs: identifying Differentially Methylated Regions between unique samples using array based methylation profiles
Source: BMC Bioinformatics. 2014 May 15;15:141. doi: 10.1186/1471-2105-15-141 (PMC4046028; doi:10.1186/1471-2105-15-141)
Supplement: Additional file 2 — DMRforPairs output for the comparison of A431-MCF7 and NA17018-NA17105. Please start from the HTML files in each folder. Available via the BMC Bioinformatics website. [file 1471-2105-15-141-S2.zip › 1394847754114233_MOESM2_ESM/A431_MCF7/figures/7204.pdf]

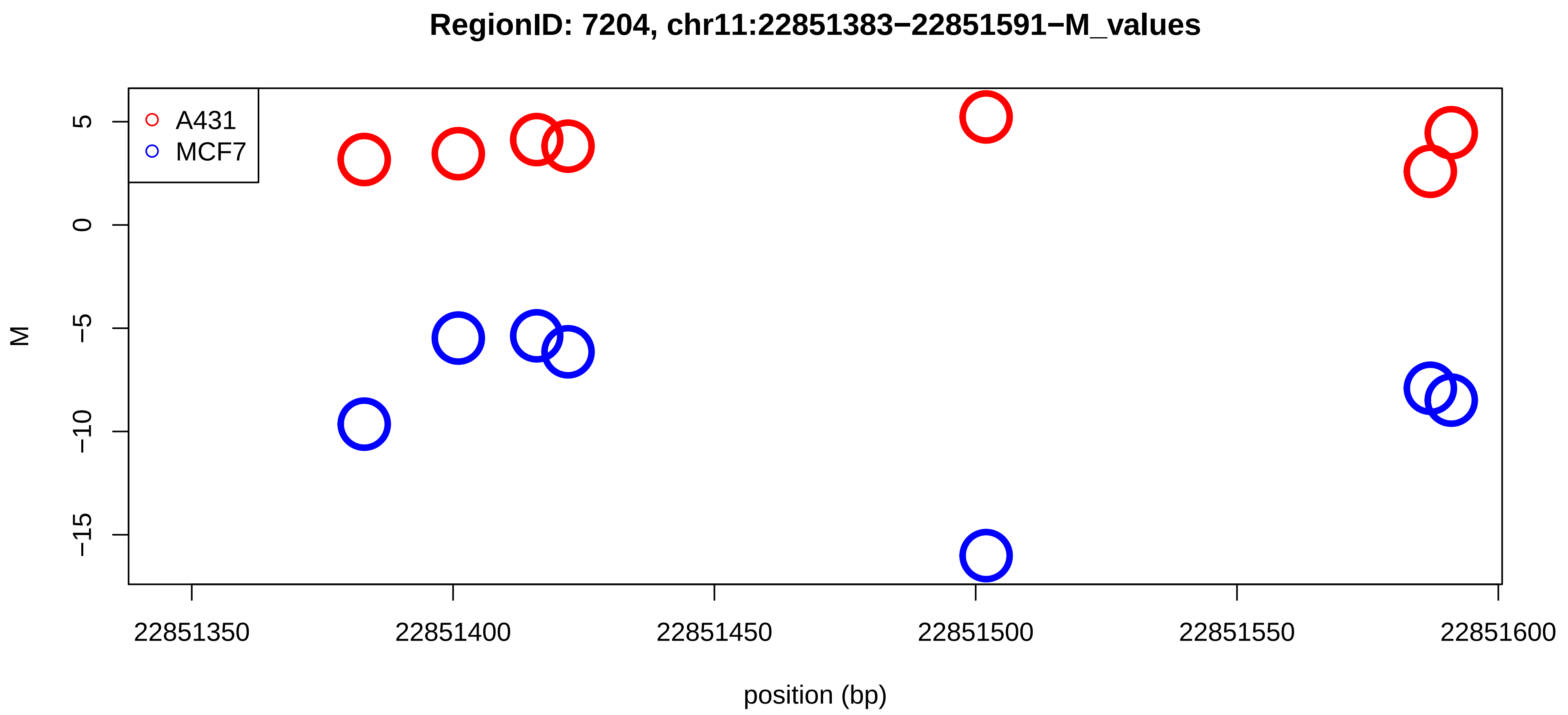
RegionID: 7204, chr11:22851383−22851591−M_values
A431
MCF7
M
5
0
−5
−10
−15
22851350
22851400
22851450
22851500
22851550
22851600
position (bp)

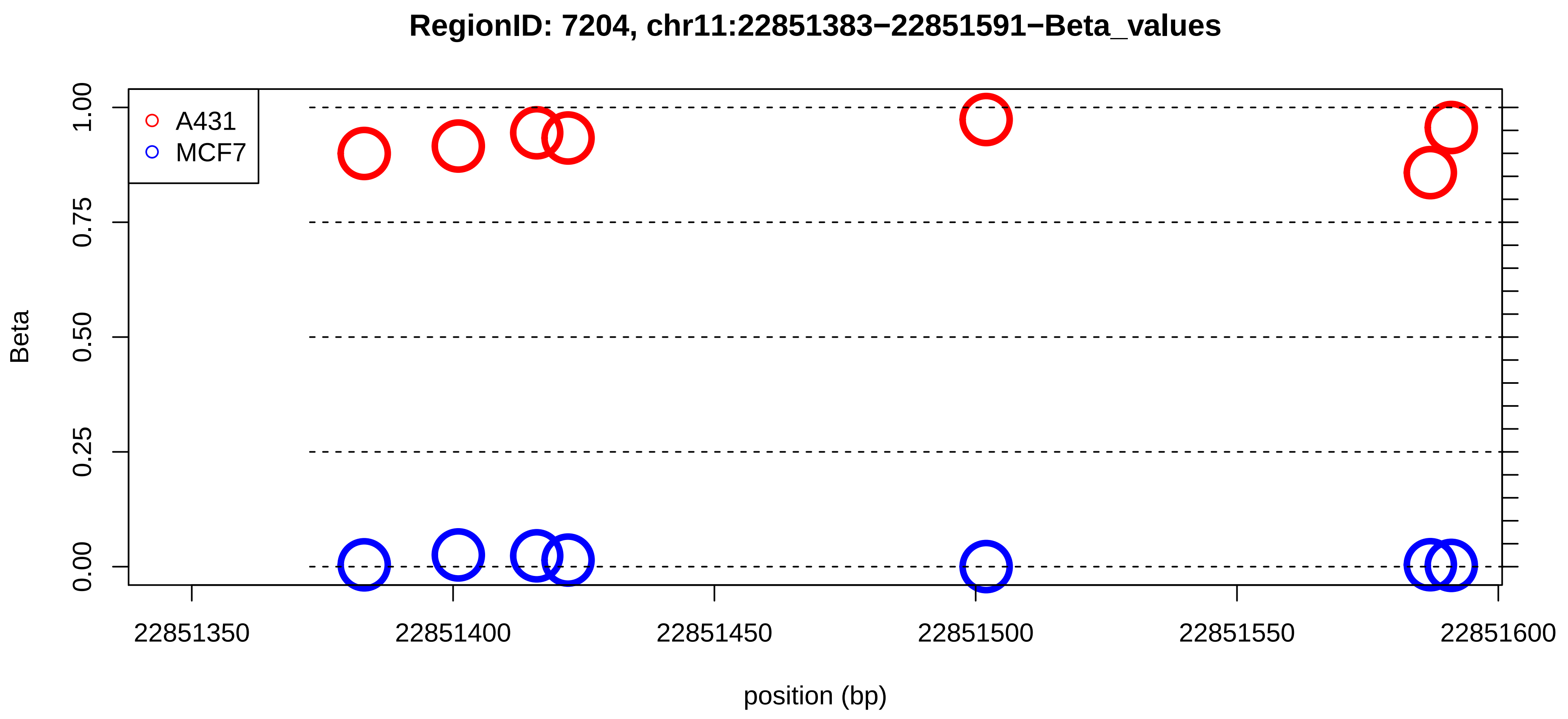
RegionID: 7204, chr11:22851383−22851591−Beta_values
A431
MCF7
Beta
1.00
0.75
0.50
0.25
0.00
22851350
22851400
22851450
22851500
22851550
22851600
position (bp)

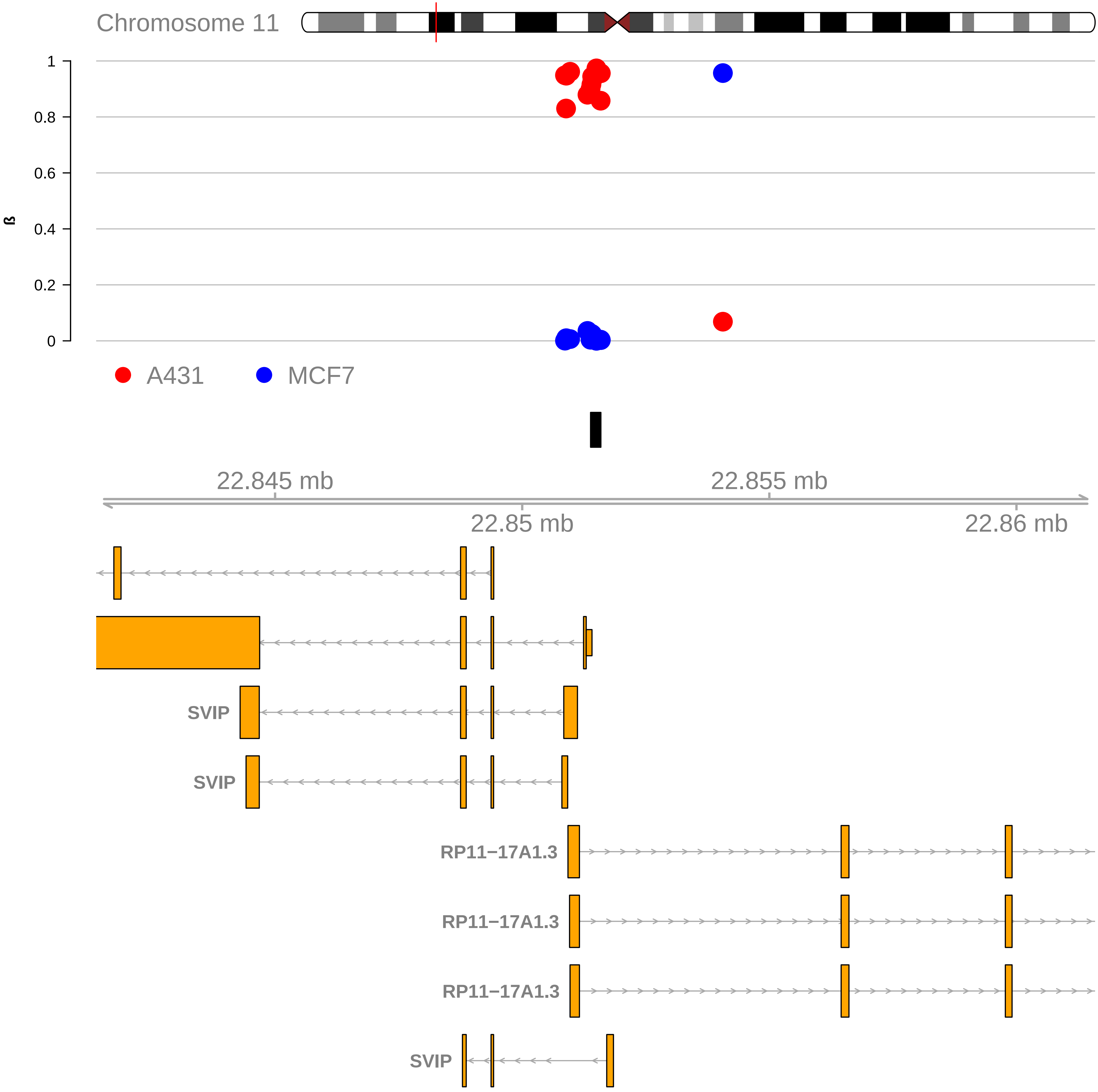

Chromosome 11
1
0.8
0.6
0.4
0.2
0
ß
A431
MCF7
22.845 mb
22.855 mb
22.85 mb
22.86 mb
SVIP
SVIP
RP11−17A1.3
RP11−17A1.3
RP11−17A1.3
SVIP
